# Draft Genome Sequences of Six Corynebacterium ulcerans Strains Isolated from Humans and Animals in Austria, 2013 to 2019

**DOI:** 10.1128/MRA.00946-20

**Published:** 2020-09-03

**Authors:** Justine Schaeffer, Steliana Huhulescu, Anna Stoeger, Franz Allerberger, Werner Ruppitsch

**Affiliations:** a Institute for Medical Microbiology and Hygiene, Austrian Agency for Health and Food Safety, Vienna, Austria; b EUPHEM Fellowship, European Centre for Disease Prevention and Control (ECDC), Stockholm, Sweden; University of Rochester School of Medicine and Dentistry

## Abstract

Corynebacterium ulcerans is an emerging pathogen responsible for severe diseases in humans and animals. Here, we present the draft genome of six C. ulcerans strains isolated in Austria. These draft genomes have 2,446,822 to 2,551,141 bp encoding 57 to 60 RNAs.

## ANNOUNCEMENT

Corynebacterium ulcerans has the ability to acquire the diphtheria toxin (DT) gene through corynebacteriophage transduction and therefore can cause diphtheria in humans (1, 2). Some C. ulcerans strains have also been shown to produce other dermonecrotic toxins (3). In Austria, diphtheria is a rare disease, with only two clinical cases documented in the last decade (4).

So far, only 17 complete and 13 draft genomes of C. ulcerans are publicly available in the National Center for Biotechnology Information (NCBI) database (https://www.ncbi.nlm.nih.gov/genome/). Increasing the number of available sequences would support molecular epidemiology studies, therapeutic and vaccine development, and also research projects investigating the mechanisms of C. ulcerans carriage, transmission, and pathogenesis.

Beginning in 2011, six C. ulcerans strains were isolated in Austria by the National Reference Center (NRC) for Diphtheria (Table 1). Data used in this study fall within the mandate given to the NRC by the Austrian Ministry of Health and do not require additional ethical approval. Bacteria were cultured on tellurite agar (Hoyle) for 24 h at 37°C. Colonies were typed using the API Coryne biochemical test (bioMérieux, Marcy-l’Étoile, France). DT production was assessed by reverse transcriptase quantitative PCR (RT-qPCR) targeting SubA and SubB of the DT (5). For sequencing, frozen isolates were plated onto Columbia agar plates with 5% sheep blood (bioMérieux) and cultured for 24 h at 37°C. Genomic DNA isolation, whole-genome sequencing (WGS), assembly, and contig filtering were performed as described previously (6). High-molecular-weight (HMW) DNA was isolated from blood cultures using the MagAttract HMW DNA kit (Qiagen, Hilden, Germany), following the manufacturer’s protocol for Gram-positive bacteria. Ready-to-sequence libraries were obtained with a NexteraXT kit (Illumina, Inc., San Diego, CA, USA). Paired-end sequencing (2 × 300 bp) was performed on a MiSeq instrument (Illumina, Inc.). The raw reads were quality controlled using FastQC v0.11.9. Trimmomatic v0.36 was used to remove adapter sequences and to trim the last 10 bp of each sequence and sequences with a quality score of <20. The reads were assembled using SPAdes v3.11.1 (7). Contigs were filtered for a minimum coverage of 5× and a minimum length of 200 bp using SeqSphere+ v6.0.0 (Ridom GmbH, Würzburg, Germany). Default parameters were used for all software. Sequencing generated 852,712 to 1,256,314 reads and a mean coverage of 75× to 110×. The NCBI Prokaryotic Genome Automatic Annotation pipeline identified 2,150 to 2,324 coding sequences, 22 to 293 pseudogenes, 5 to 8 rRNA genes, and 49 to 52 tRNA genes (Table 1).

**TABLE 1:** Characteristics, sequencing data, and accession numbers of C. ulcerans isolates

Characteristic^*a*^	Data for strain^*b*^:					
	02-13	04-13	05-13	04-15	06-16	06-19
Collection date (day/mo/yr)	21/3/2013	23/5/2013	23/5/2013	18/3/2015	14/12/2016	08/5/2019
Sample type	Human wound	Animal	Animal	Human wound	Human wound	Human wound
Tox	+	+	+	−	+	−
ST	332	578	578	339	565	339
Patient age (yr)	84			63	81	81
Patient sex	F			M	M	F
Symptoms	Diabetes			Ulcus cruris	Wound	Infected wound
Austrian city of isolation	Linz	Mödling	Mödling	Krems	Wien	Wien
Genome size (Mbp)	2.6	2.6	2.6	2.5	2.6	2.5
No. of reads	2,182,590	2,386,014	1,361,822	2,008,938	1,052,652	1,999,384
Total no. of genes	2,316	2,378	2,384	2,209	2,322	2,210
No. of RNAs	59	57	60	59	60	58
Avg coverage (×)	187	202	190	123	110	180
No. of contigs	20	13	18	15	20	19
Contig *N*_50_ (bp)	304,752	328,902	325,464	395,442	281,303	385,889
G+C content (%)	53.17	54.27	54.26	53.11	53.29	53.19
GenBank accession no.	JABGCM000000000	JABGCN000000000	JABGCO000000000	JABGCP000000000	JABGCQ000000000	JABGCR000000000
SRA accession no.	SRR11485668	SRR11485667	SRR11485666	SRR11485665	SRR11485664	SRR11485663

a Tox, toxigenic; ST, sequence type.

b F, female; M, male; +, yes; −, no.

Antibiotic resistance genes were searched for using the ResFinder tool available from the Center for Genomic Epidemiology, and none were found (8). Multilocus sequence typing (MLST) was performed by extracting data from the WGS data using SeqSphere+. An *ad hoc* core genome MLST (cgMLST) scheme comprising 1,215 targets was established using strain BR-AD22 (GenBank accession no. NC_015683.1) as a reference (SeqSphere+ v6.0.0 with default settings). All isolates had cgMLST targets that were >92% good.

Four Austrian strains were isolated from patients with skin infections, and two from animals. Among them, 4/6 produced diphtheria toxins. The two animal strains (04-13 and 05-13) belonged to sequence type 578 (ST578) but showed 308 allelic differences. Strain 04-13 was closely related to reference strain KL1196 (accession no. SDVD00000000), isolated from a German deer in 2018, with 16 allelic differences. Two human isolates belonged to ST339 (04-15 and 06-19) and differed by 8 alleles. The other human isolates belonged to ST332 (02-13) and ST565 (06-16), differing by 110 alleles in their core genome. No GenBank isolate was closely related to these two Austrian isolates. This underlines the lack of representativeness of C. ulcerans diversity by its few publicly available sequences.

Comparison of these six C. ulcerans genomes showed an important variability between strains. This underlines the need for more sequence data in order to fully cover C. ulcerans diversity and produce a reliable framework for future studies.

### Data availability.

This whole-genome shotgun project has been deposited in DDBJ/ENA/GenBank under accession no. JABGCM000000000 (02-13), JABGCN000000000 (04-13), JABGCO000000000 (05-13), JABGCP000000000 (04-15), JABGCQ000000000 (06-16), and JABGCR000000000 (06-19). The versions described in this paper are the first versions, JABGCM010000000 through JABGCR010000000. The raw sequence reads have been deposited in the Sequence Read Archive (SRA) under accession no. SRR11485668 (02-13), SRR11485667 (04-13), SRR11485666 (05-13), SRR11485665 (04-15), SRR11485664 (06-16), and SRR11485663 (06-19).
